# Construction and Analysis of Double Helix for Triangular Bipyramid and Pentangular Bipyramid

**DOI:** 10.1155/2020/5609593

**Published:** 2020-05-14

**Authors:** Tao Deng

**Affiliations:** ^1^Key Laboratory of China's Ethnic Languages and Information Technology of Ministry of Education, Northwest Minzu University, Lanzhou 730030, China; ^2^Key Laboratory of Streaming Data Computing Technologies and Application, Northwest Minzu University, Lanzhou 730030, China; ^3^School of Mathematics and Computer Science, Northwest Minzu University, Lanzhou 730030, China

## Abstract

DNA cages can be joined together to make larger 3D nanostructures on which molecular electronic circuits and tiny containers are built for drug delivery. The mathematical models for these promising nanomaterials play important roles in clarifying their assembly mechanism and understanding their structures. In this study, we propose a mathematical and computer method to construct permissible topological structures with double-helical edges for a triangular bipyramid and pentangular bipyramid. Furthermore, we remove the same topological links, without eliminating the nonrepeated ones for a triangular bipyramid and pentangular bipyramid. By analyzing characteristics of these unique links, some self-assembly and statistic rules are discussed. This study may obtain some new insights into the DNA assembly from the viewpoint of mathematics, promoting the comprehending and design efficiency of DNA polyhedra with required topological structures.

## 1. Introduction

The polyhedra made up of several triangles possess stable structures. Of five Platonic solids constructed from one type of polygon, three are made of triangles, accounting for 60%. Of thirteen Archimedes solids formed by two or three polygons, nine are composed of triangles, accounting for 69.2%. The Biosphere for the 1967 World Fair, Expo 67 surprised the world, for it reached its maximum volume by using minimum materials. The stress of the geodesic domes is distributed by triangles, having stable nature, formed by crossed geodesics. In fact, geodesic domes are based on deltahedra which refer to polyhedra constructed from triangular faces. The deltahedra include eight ones in regular and semiregular polyhedra of high symmetry: (1) regular tetrahedron; (2) triangular bipyramid; (3) regular octahedron; (4) pentangular bipyramid; (5) trigonal dodecahedron; (6) tritetrakis triprism; (7) ditetrakis tetragonal antiprism; and (8) icosahedron. Of these polyhedra, (1), (3), and (8) are regular polyhedra and the other five are semiregular polyhedra.

DNA is an attractive engineering material because strands with complementary base sequences recognize and bind to each other, enabling complex molecular structures to be made by self-assembly. DNA has been used as an ideal material in 3D nanostructures for its good self-assembly ability [1, 2]. Among DNA deltahedra, synthesis and application have been done regarding the tetrahedron [3, 4], the triangular bipyramid [5, 6], the octahedron [7, 8], and the icosahedron [9]. DNA deltahedra-like nanostructures have played even more important roles in drug delivery and imaging probes [3, 4, 6, 7, 9–11]. Furthermore, DNA nanomaterials will find a wide range of applications in biosensing, bioimaging, and biomedicine due to their intriguing structures and functions [12]. In the meanwhile, some researchers are trying to study the design strategies for DNA cages from the mathematical and interdisciplinary perspective. Benson et al. proposed a general method to construct scaffolded DNA nanostructures from flat sheet meshes with the computer-aided technique [13]. Qiu and his colleagues took a mathematical approach to answer the complex question of what models are suitable to describe the DNA polyhedron structure [14–19]. Guo et al. provided a topological approach to assemble DNA tetrahedra and DNA triangular prism [20, 21]. Lin et al. obtained all permissible topological structures for a DNA trigonal bipyramid with double-helical edges [22], and they took into account mirror images of twists regarding the building blocks for constructing trigonal bipyramid links and calculated the exact number for permissible DNA links. In the previous study, we constructed and analyzed three Archimedean solids from the point of view of mathematics and computation program [23]. Archimedean solids are of high symmetry and own a large number of DNA polyhedral links. In consideration of significance of deltahedra-like nanostructures with lower symmetry, the triangular bipyramid and pentangular bipyramid assembled by a double helix as well as their nonrepeated isotopic links are studied. We start by introducing the construction rules of the vertex and edge for double-helical structures and then provide the construction algorithm and analytical method for the triangular bipyramid and pentangular bipyramid. Unlike polyhedra with high symmetry, the triangular bipyramid and pentangular bipyramid could not get their projections which own the uncrossed line. By introducing and analyzing their space diagrams, the nonrepeated triangular bipyramid and pentangular bipyramid are obtained. In this process, some statistical law and assembly mechanism for deltahedra are discussed. This study may promote the comprehending and design efficiency of DNA polyhedra with required topological structures.

## 2. Methods

### 2.1. DNA Polyhedral Links

A theoretical framework for characterizing DNA cages is a polyhedral link which turns the double-stranded DNA molecule into a computable topological model [24]. In fact, a polyhedral link is an interlinked and interlocked architecture obtained from a polyhedral graph *G*, by using branchedjunctions and twisted lines to replace the vertices and edges [14]. To fully understand this study, we have to explain some basic concepts used in our previous work. The crossing number of a polyhedral link *c* is the least number of crossings that occur in any projection of the polyhedral link. The component number of a polyhedral link *μ* is the number of loops (rings) knotted with each other [15]. These parameters brought some topological viewpoints to describe and answer structural characteristics of DNA polyhedra [18, 19].

**Figure 1:**
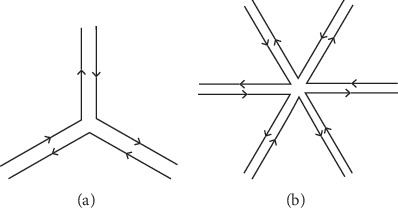
Building motifs of three- and five-branched junctions.

In this study, star-like motifs appear in stable structures of artificial DNA cages and virus capsids. The blocks of three- and five-branched junctions, shown in Figure 1, are located on the three-degree vertices and the five-degree ones, respectively. The triangular bipyramid and pentangular bipyramid just have the structure of vertex like these. Note that the arrows indicate the directions of DNA strands and the two strands are parallel and oppositely oriented.

**Figure 2:**
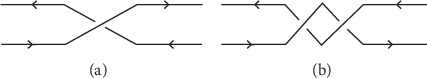
One crossing (a half-twist) and two crossings (a twist).

Also in the polyhedral link, there are either odd crossings or even crossings on each edge. We consider both the case of an odd number of crossings and the case of an even number of crossings. For simplicity, one crossing (a half-twist) and two crossings (a twist) represent an odd number of crossings and an even number of crossings, respectively, as shown in Figure 2. We use branch-point vertex and odd-even-crossing edge structures, together with some rule in the assembly of DNA strands to derive a construction algorithm for the triangular bipyramid and pentangular bipyramid.

### 2.2. Construction Rule

Polyhedral links include DNA polyhedral links and non-DNA polyhedral links, and the former could satisfy the rule in which the two strands are antiparallel, but the latter could not. Jonoska et al. revealed that the minimal number of circular strands needed to construct DNA cages is 2 given that every face of the polyhedron must have an even number of crossings [25, 26]. Above all, we need to list all the types satisfying this construction rule. Actually, the rule turns to be the generate-and-test paradigm. Therefore, the triangular bipyramid and the pentangular bipyramid are studied in terms of artificial DNA molecules, and mathematical characteristics and construction parameters for the double-helical structure are explored by analyzing the result of computer programs.

### 2.3. Triangular Bipyramid

**Figure 3:**
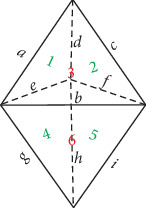
Space diagram of a triangular bipyramid with labels.

The space diagram of a triangular bipyramid is shown in Figure 3. It has five vertices (*V* = 5), and its nine edges (*E* = 9) and six faces (*F* = 6) are indicated by *a*–*i* and 1–6, respectively. Note that Faces 3 and 6 (denoted by red numbers) are outward, while Faces 1, 2, 4, and 5 (denoted by green numbers) are inward. The sum of the crossings on each face equals *a* + *d* + *e*, *c* + *d* + *f*, *a* + *b* + *c*, *e* + *g* + *h*, *f* + *h* + *i*, and *b* + *g* + *i*. The values of *a*, *b*, *c*, *d*, *e*, *f*, *g*, *h*, and *i* take 1 or 2, which means that either one crossing or two crossings occur on each edge. Most importantly, each face of the triangular bipyramid must have an even number of crossings. To construct the corresponding DNA triangular bipyramids is to give a program which goes through all the cases satisfying the above criteria. The result specifies the constructing parameters for the corresponding DNA triangular bipyramid links.

### 2.4. Pentangular Bipyramid

**Figure 4:**
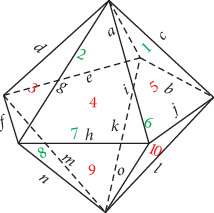
Space diagram of a pentangular bipyramid with labels.

A pentangular bipyramid has seven vertices (*V* = 7), fifteen edges (*E* = 15), and ten faces (*F* = 10).  Figure 4 shows its space diagram as well as labels, in which its nine edges and six faces are indicated by *a*–*o* and 1–10, respectively. The sums of the crossing number on each face equal *a* + *b* + *c*, *a* + *d* + *e*, *d* + *f* + *g*, *g* + *h* + *i*, *c* + *i* + *j*, *b* + *k* + *l*, *e* + *k* + *m*, *f* + *m* + *n*, *h* + *n* + *o*, and *j* + *l* + *o*. Similarly, one crossing and two crossings represent an odd number of crossings and even number of crossings, respectively. The values of *a*, *b*, *c*, *d*, *e*, *f*, *g*, *h*, and *i* take 1 or 2, which means that either one crossing or two crossings occur on each edge.

### 2.5. Algorithm and Program

Here, a Python program is presented for obtaining parameters for the triangular bipyramid links. In this program, *a*–*i* represents the edges of the triangular bipyramid, while the parameter *x* is specially set to count the total number of odd-crossing edges. This program could also be applied to the pentangular bipyramid crossings = [1,2].

  for *a* in crossings:
   for *d* in crossings:
   for *e* in crossings:
     if (*a* + *d* + *e*)%2 = = 0: #The sum of the crossings within Face 1 must be an even number
       for *c* in crossings:
     for *f* in crossings:
     if (*c* + *d* + *f*)%2 = = 0: #The sum of the crossings within Face 2 must be an even number
       for *b* in crossings:
      if (*a* + *b* + *c*)%2 = = 0: #The sum of the crossings within Face 3 must be an even number
         for *g* in crossings:
       for *h* in crossings:
        if (*e* + *g* + *h*)%2 = = 0: #The sum of the crossings within Face 4 must be an even number
           for *i* in crossings:
         if (*f* + *h* + *i*)%2 = = 0: #The sum of the crossings within Face 5 must be an even number
            if (*b* + *g* + *i*)%2 = = 0: #The sum of the crossings within Face 6 must be an even number
          set = [*a*, *b*, *c*, *d*, *e*, *f*, *g*, *h*]
          *x* = set.count(1) #The total number of the odd-crossing edges
          print (‘,'.join([“{} = {}”.format(‘abcdefghx'[*i*], *x*) for *i*, *x* in enumerate(set + [*x*])]))

## 3. Results and Discussion

### 3.1. Triangular Bipyramid

The number of types obtained by the program is 16 (2^4^).  Table 1 lists the parameters for 16 types of triangular bipyramids made by DNA strands computed from the program. Note that “even” denotes the number of even-crossing edges which is the difference between the number of edges (9 for a triangular bipyramid) and the number of odd-crossing edges. The triangular bipyramid, unlike polyhedra with high symmetry [23], could not get a projection having an uncrossed line due to its low symmetry. Therefore, a space diagram is required to describe the triangular bipyramid. By drawing the space diagram of triangular bipyramids according to the data in Table 1, the number of strands to construct triangular bipyramids is obtained. The DNA strand corresponds to the link component in topology. So the strand number actually equals the component number.  Table 1 also specifies the case of strands. Compared with the polyhedra of high symmetry such as the truncated tetrahedron [23], the potential type for the triangular bipyramid is smaller.

**Figure 5:**
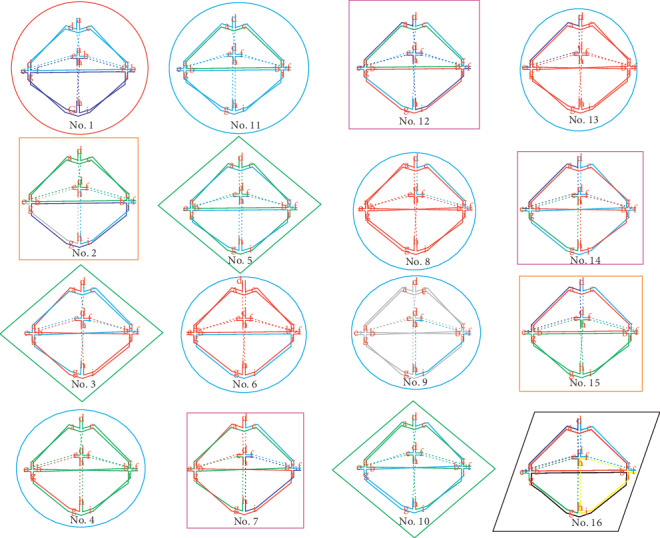
Classifying 16 triangular bipyramids into six types.

However, there are some equivalent ones in all 16 types derived from the program, for some links turn out to be the same one by rotation. Two links are ambient isotopic which means that two links have exactly the same topological structure or can overlap with each other by rotation [22]. Isotopic links are the sufficient condition for links with the same components. So links having different components must be distinct from each other. Classifying the links by the component number, we obtain three groups: two components (A), four components (B), and six components (C). Comparison of the space diagrams demonstrates that there are a total of six unique types of 16 triangular bipyramids and links in the same type are actually isotopic.  Figure 5 shows the classifying process of 16 triangular bipyramids. Note that triangular bipyramids in the same shape and color are isotopic.

**Figure 6:**
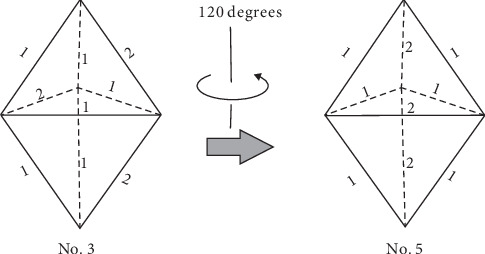
Change of equivalent links.

Specifically, there are three distinct types in group A, Type I: No. 1; Type II: No. 3, No. 5, and No. 10; and Type III: No. 4, No. 6, No. 8, No. 9, No. 11, and No. 13. Also, there are two distinct types in group B, Type IV: No. 2 and No. 15 and Type V: No. 7, No. 12, and No. 14. Only No. 16 is in group C, Type VI.  Figure 6 shows the two isotopic links of triangular bipyramids, in which link No. 3 turns to be link No. 5 by 120 degrees' rotation.

Therefore, there are 6 unique types of DNA polyhedral links for the triangular bipyramid, which are characterized in Table 2. The parameters *μ* and *c* indicate the component (strand) number and crossing number, respectively [19]. “*X* odd and *Y* even” means that this type of polyhedral link has *X* edges of odd crossings and *Y* ones of even crossings.

**Figure 7:**
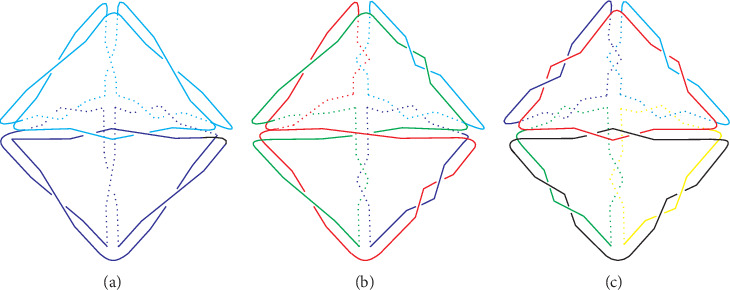
Space diagram of three DNA triangular bipyramids (3^6^): (a) *μ* = 2, 6 odd and 3 even (Type I); (b) *μ* = 4, 4 odd and 5 even (Type V); (c) *μ* = 6, 0 odd and 9 even (Type VI).

As we can see from Table 1 and Table 2, the triangular bipyramid has a small number of types even though it owns six faces. This may be due to that the triangular bipyramid has two types of degrees of vertices—3 and 4, and it owns low symmetry. The triangular bipyramid owns an even number of components: two, four, and six components. Of 6 types of DNA triangular bipyramids, three have two components or strands, accounting for about 50%, while two have four ones, accounting for 33%, and only one has six ones, accounting for 17%. This shows that the probability of the unique DNA triangular bipyramid is high with the component number reaching the median value of the face number. Two polyhedral links with the same number of components always have different numbers of even- and odd-crossing edges. Intuitively, with the increase of even-crossing edges, the double-helical polyhedral link owns more circular strands or components. These conclusions are tested in the part of the pentangular bipyramid.  Figure 7 shows the space diagrams of three unique DNA triangular bipyramids in Table 2.

### 3.2. Pentangular Bipyramid

The types and number of resulting DNA pentangular bipyramids are obtained using the program above.  Table 3 lists 64 types of pentangular bipyramids made by DNA strands.

The pentangular bipyramid, like the triangular bipyramid, could not get a projection having uncrossed lines because of its low symmetry. Similarly, by drawing the space diagram of pentangular bipyramids according to the data in Table 3, the component number, even-crossing edge number (the number of edges of even crossings), and odd-crossing edge number (the number of edges of odd crossings) of DNA polyhedral links for pentangular bipyramids are obtained. Classifying the links by the component number, we obtain five groups: two components (A), four components (B), six components (C), eight components (D), and ten components (E). Comparison of the space diagrams demonstrates that there are a total of twelve unique types of 64 triangular bipyramids and links in the same type are actually isotopic. Specifically, there are three nonequivalent types in group A, Type I: No. 2, No. 10, No. 12, No. 14, No. 26, No. 33, No. 35, No. 37, No. 41, and No. 49; Type II: No. 7, No. 17, No. 21, No. 27, and No. 40; and Type III: No. 23. Also, there are four nonequivalent types in group B, Type IV: No. 1, No. 11, No. 25, No. 34, and No. 38; Type V: No. 4, No. 6, No. 20, No. 30, No. 32, No. 43, No. 45, No. 53, No. 55, and No. 59; Type VI: No. 5, No. 19, No. 29, No. 44, and No. 56; and Type VII: No. 8, No. 16, No. 18, No. 22, No. 28, No. 39, No. 47, No. 51, No. 57, and No. 61. There are three nonequivalent types in group C, Type VIII: No. 3, No. 31, No. 46, No. 54, and No. 60; Type IX: No. 9, No. 13, No. 36, No. 42, and No. 50; and Type X: No. 24 and No. 63. There is only one nonequivalent type in group D, Type XI, including No. 15, No. 48, No. 52, No. 58, and No. 62. Only No. 64 is in group E, Type XII. Supplementary materials (available (here)) contain the DNA strand classification for the pentangular bipyramid, which shows the process of identifying isotopic links for the pentangular bipyramid.  Table 4 characterizes 12 unique types of DNA polyhedral links for pentangular bipyramids, in which *μ* and *c* indicate the component number and crossing number, respectively.

**Figure 8:**
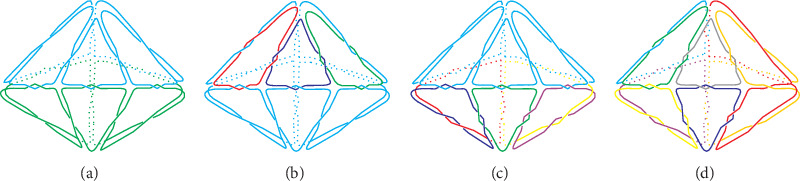
Space diagram of four DNA pentangular bipyramids (3^10^): (a) *μ* = 2, 10 odd and 5 even (Type III); (b) *μ* = 4, 7 odd and 8 even (Type VII); (c) *μ* = 6, 5 odd and 10 even (Type X); (d) *μ* = 8, 4 odd and 11 even (Type XI).

As shown in Table 4, pentangular bipyramids possess an even number of components—2, 4, 6, 8, and 10—due to their even-numbered faces. Of twelve types of DNA pentangular bipyramids, three have two components, accounting for 25%; four have four ones, accounting for 33.3%; three have six ones, accounting for 25%; one has eight ones, accounting for 8.33%; and one has ten ones, accounting for 8.33%. Similarly, the number of distinct DNA pentangular bipyramids reaches the highest when the component number approaches the median value of the face number.  Figure 8 shows the space diagram of four DNA pentangular bipyramids.

## 4. Conclusion

1. The triangular bipyramid and pentangular bipyramid belong to deltahedra, made up of triangular faces. The results demonstrate that the number of types of certain polyhedra could be obtained by the computer program in the case that each face of the polyhedron has an even number of crossings.
2. The number of potential types of triangular bipyramids and pentangular bipyramids is 16 and 64, respectively. However, only 6 types of triangular bipyramids and 12 ones of pentangular bipyramids remain unique by removing the same types.
3. The number of components *μ* must be even and cannot be odd. The component number *μ* increases from 2 to the number of faces. Most DNA polyhedral links own lower symmetry. Only when its component number equals its face number, the polyhedral link has original symmetry.
4. Even two polyhedral links have the same number of components, and they always have different numbers of even- and odd-crossing edges, and vice versa. In most cases, the more the circular strands or components a DNA polyhedral link has, the more the even-crossing edges it has.
5. With the decrease of symmetry, the polyhedra may own less potential DNA links. Unique types of DNA links peak with the component number approaching the median value of the face number.

## Figures and Tables

**Table 1:** Parameters for 16 types of DNA triangular bipyramids.

No.	*a*	*b*	*c*	*d*	*e*	*f*	*g*	*h*	*i*	Odd	Even	Strand
1	1	2	1	1	2	2	1	1	1	6	3	2
2	1	2	1	1	2	2	2	2	2	3	6	4
3	1	1	2	1	2	1	1	1	2	6	3	2
4	1	1	2	1	2	1	2	2	1	5	4	2
5	1	2	1	2	1	1	1	2	1	6	3	2
6	1	2	1	2	1	1	2	1	2	5	4	2
7	1	1	2	2	1	2	1	2	2	4	5	4
8	1	1	2	2	1	2	2	1	1	5	4	2
9	2	1	1	1	1	2	1	2	2	5	4	2
10	2	1	1	1	1	2	2	1	1	6	3	2
11	2	2	2	1	1	1	1	2	1	5	4	2
12	2	2	2	1	1	1	2	1	2	4	5	4
13	2	1	1	2	2	1	1	1	2	5	4	2
14	2	1	1	2	2	1	2	2	1	4	5	4
15	2	2	2	2	2	2	1	1	1	3	6	4
16	2	2	2	2	2	2	2	2	2	0	9	6

**Table 2:** Characterization of 6 unique types of DNA triangular bipyramids.

Type	*μ*	*c*	Notes
I	2	12	6 odd and 3 even
II	2	12	6 odd and 3 even
III	2	13	5 odd and 4 even
IV	4	15	3 odd and 6 even
V	4	14	4 odd and 5 even
VI	6	18	0 odd and 9 even

**Table 3:** 64 types of DNA pentangular bipyramids.

No.	*a*	*b*	*c*	*d*	*e*	*f*	*g*	*h*	*i*	*j*	*k*	*l*	*m*	*n*	*o*	Odd	Even	Strand
1	1	1	2	1	2	1	2	1	1	1	1	2	1	2	1	10	5	4
2	1	1	2	1	2	1	2	1	1	1	2	1	2	1	2	9	6	2
3	1	1	2	1	2	1	2	2	2	2	1	2	1	2	2	6	9	6
4	1	1	2	1	2	1	2	2	2	2	2	1	2	1	1	7	8	4
5	1	1	2	1	2	2	1	1	2	2	1	2	1	1	2	8	7	4
6	1	1	2	1	2	2	1	1	2	2	2	1	2	2	1	7	8	4
7	1	1	2	1	2	2	1	2	1	1	1	2	1	1	1	10	5	2
8	1	1	2	1	2	2	1	2	1	1	2	1	2	2	2	7	8	4
9	1	1	2	2	1	1	1	1	2	2	1	2	2	1	2	8	7	6
10	1	1	2	2	1	1	1	1	2	2	2	1	1	2	1	9	6	2
11	1	1	2	2	1	1	1	2	1	1	1	2	2	1	1	10	5	4
12	1	1	2	2	1	1	1	2	1	1	2	1	1	2	2	9	6	2
13	1	1	2	2	1	2	2	1	1	1	1	2	2	2	1	8	7	6
14	1	1	2	2	1	2	2	1	1	1	2	1	1	1	2	9	6	2
15	1	1	2	2	1	2	2	2	2	2	1	2	2	2	2	4	11	8
16	1	1	2	2	1	2	2	2	2	2	2	1	1	1	1	7	8	4
17	1	2	1	1	2	1	2	1	1	2	1	1	1	2	1	10	5	2
18	1	2	1	1	2	1	2	1	1	2	2	2	2	1	2	7	8	4
19	1	2	1	1	2	1	2	2	2	1	1	1	1	2	2	8	7	4
20	1	2	1	1	2	1	2	2	2	1	2	2	2	1	1	7	8	4
21	1	2	1	1	2	2	1	1	2	1	1	1	1	1	2	10	5	2
22	1	2	1	1	2	2	1	1	2	1	2	2	2	2	1	7	8	4
23	1	2	1	1	2	2	1	2	1	2	1	1	1	1	1	10	5	2
24	1	2	1	1	2	2	1	2	1	2	2	2	2	2	2	5	10	6
25	1	2	1	2	1	1	1	1	2	1	1	1	2	1	2	10	5	4
26	1	2	1	2	1	1	1	1	2	1	2	2	1	2	1	9	6	2
27	1	2	1	2	1	1	1	2	1	2	1	1	2	1	1	10	5	2
28	1	2	1	2	1	1	1	2	1	2	2	2	1	2	2	7	8	4
29	1	2	1	2	1	2	2	1	1	2	1	1	2	2	1	8	7	4
30	1	2	1	2	1	2	2	1	1	2	2	2	1	1	2	7	8	4
31	1	2	1	2	1	2	2	2	2	1	1	1	2	2	2	6	9	6
32	1	2	1	2	1	2	2	2	2	1	2	2	1	1	1	7	8	4
33	2	1	1	1	1	1	2	1	1	2	1	2	2	1	2	9	6	2
34	2	1	1	1	1	1	2	1	1	2	2	1	1	2	1	10	5	4
35	2	1	1	1	1	1	2	2	2	1	1	2	2	1	1	9	6	2
36	2	1	1	1	1	1	2	2	2	1	2	1	1	2	2	8	7	6
37	2	1	1	1	1	2	1	1	2	1	1	2	2	2	1	9	6	2
38	2	1	1	1	1	2	1	1	2	1	2	1	1	1	2	10	5	4
39	2	1	1	1	1	2	1	2	1	2	1	2	2	2	2	7	8	4
40	2	1	1	1	1	2	1	2	1	2	2	1	1	1	1	10	5	2
41	2	1	1	2	2	1	1	1	2	1	1	2	1	2	1	9	6	2
42	2	1	1	2	2	1	1	1	2	1	2	1	2	1	2	8	7	6
43	2	1	1	2	2	1	1	2	1	2	1	2	1	2	2	7	8	4
44	2	1	1	2	2	1	1	2	1	2	2	1	2	1	1	8	7	4
45	2	1	1	2	2	2	2	1	1	2	1	2	1	1	2	7	8	4
46	2	1	1	2	2	2	2	1	1	2	2	1	2	2	1	6	9	6
47	2	1	1	2	2	2	2	2	2	1	1	2	1	1	1	7	8	4
48	2	1	1	2	2	2	2	2	2	1	2	1	2	2	2	4	11	8
49	2	2	2	1	1	1	2	1	1	1	1	1	2	1	2	9	6	2
50	2	2	2	1	1	1	2	1	1	1	2	2	1	2	1	8	7	6
51	2	2	2	1	1	1	2	2	2	2	1	1	2	1	1	7	8	4
52	2	2	2	1	1	1	2	2	2	2	2	2	1	2	2	4	11	8
53	2	2	2	1	1	2	1	1	2	2	1	1	2	2	1	7	8	4
54	2	2	2	1	1	2	1	1	2	2	2	2	1	1	2	6	9	6
55	2	2	2	1	1	2	1	2	1	1	1	1	2	2	2	7	8	4
56	2	2	2	1	1	2	1	2	1	1	2	2	1	1	1	8	7	4
57	2	2	2	2	2	1	1	1	2	2	1	1	1	2	1	7	8	4
58	2	2	2	2	2	1	1	1	2	2	2	2	2	1	2	4	11	8
59	2	2	2	2	2	1	1	2	1	1	1	1	1	2	2	7	8	4
60	2	2	2	2	2	1	1	2	1	1	2	2	2	1	1	6	9	6
61	2	2	2	2	2	2	2	1	1	1	1	1	1	1	2	7	8	4
62	2	2	2	2	2	2	2	1	1	1	2	2	2	2	1	4	11	8
63	2	2	2	2	2	2	2	2	2	2	1	1	1	1	1	5	10	6
64	2	2	2	2	2	2	2	2	2	2	2	2	2	2	2	0	15	10

**Table 4:** Characterization of 12 unique types of DNA pentangular bipyramids.

Type	No.	*μ*	*c*	Notes
I	1	2	21	9 odd and 6 even
II	2	2	20	10 odd and 5 even
III	3	2	20	10 odd and 5 even
IV	4	4	20	10 odd and 5 even
V	5	4	23	7 odd and 8 even
VI	6	4	22	8 odd and 7 even
VII	7	4	23	7 odd and 8 even
VIII	8	6	24	6 odd and 9 even
IX	9	6	21	9 odd and 6 even
X	10	6	25	5 odd and 10 even
XI	11	8	29	4 odd and 11 even
XII	12	10	30	0 odd and 15 even

## Data Availability

The data used to support the findings of this study could be computed through the program proposed in this article.

## Supplementary Materials

Supplementary Materials This manuscript has a supplementary file about DNA strand classification for pentangular bipyramids, which shows the process of identifying homogeneous types of pentangular bipyramids. Each of 64 links is drawn and classified by the component number (strand number). Therefore, we obtain five groups of two strands (A), four strands (B), six strands (C), and eight strands (D). Figure Two strands (pentangular bipyramid): links in this group have two strands, including No. 2, No. 7, No. 10, No. 12, No. 14, No. 17, No. 21, No. 23, No. 26, No. 27, No. 33, No. 35, No. 37, No. 40, No. 41, and No. 49. Figure Four strands (pentangular bipyramid): links in this group have four strands, including No. 1, No. 4, No. 5, No. 6, No. 8, No. 11, No. 16, No. 18, No. 19, No. 20, No. 22, No. 25, No. 28, No. 29, No. 30, No. 32, No. 34, No. 38, No. 39, No. 43, No. 44, No. 45, No. 47, No. 51, No. 53, No. 55, No. 56, No. 57, No. 59, and No. 61. Figure Six strands (pentangular bipyramid): links in this group have six strands, including No. 3, No. 9, No. 13, No. 24, No. 31, No. 36, No. 42, No. 46, No. 50, No. 54, No. 60, and No. 63. Figure Eight strands (pentangular bipyramid): links in this group have eight strands, including No. 15, No. 48, No. 52, No. 58, and No. 62. Only No. 64 is in the group of ten strands, not shown in the picture. By comparing the space diagrams above, we obtain twelve unique types of 64 triangular bipyramids and the links in the same type are actually homogeneous.
